# Renal Fibrosis and Oxidative Stress Induced by Silica Nanoparticles in Male Rats and Its Molecular Mechanisms

**DOI:** 10.5812/ijpr-143703

**Published:** 2024-03-26

**Authors:** Bakhta Aouey, Khadija Boukholda, Alin Ciobica, Vasile Burlui, Rachid Soulimani, Fatiha Chigr, Hamadi Fetoui

**Affiliations:** 1 Laboratory of Toxicology-Microbiology and Environmental Health (17ES06), Faculty of Sciences of Sfax, University of Sfax, BP1171, 3000 Sfax, Tunisia; 2 Department of Biology, Faculty of Biology, “Alexandru Ioan Cuza” University of Iasi, Bd. Carol I 20A, 700505 Iasi, Romania; 3 Center of Biomedical Research, Romanian Academy, Iasi, Romania; 4 Academy of Romanian Scientists, 3 Ilfov, 050044, Bucharest, Romania; 5 Department of Biomaterials, Faculty of Dental Medicine, Apollonia University, 700511 Iasi, Romania; 6 Neurotoxicology and Bioactivity/LCOMS, Campus Bridoux, University of Lorraine, 57070, Metz, France; 7 Biological Engineering Laboratory, Faculty of Sciences and Techniques, Sultan Moulay Slimane University, Beni Mellal, Morocco

**Keywords:** Silica Nanoparticle, Oxidative Stress, Renal Fibrosis, TGF-β1, Matrix Metalloproteinase

## Abstract

**Background:**

The utilization of amorphous silica nanoparticles (SiNPs) is gaining popularity in various applications, but it poses a potential risk to human and environmental health. However, the underlying causes and mechanisms of SiNPs-induced kidney damage are still largely unknown.

**Objectives:**

This study aimed to investigate the SiNPs-induced damage in the kidney and further explore the possible mechanisms of SiNPs-induced nephrotoxicity.

**Methods:**

Thirty adult male rats were divided into 3 different groups. Rats in groups 2 and 3 were administered SiNPs at 2 dosage levels (25 and 100 mg/kg of body weight), while the rats in the control group received no treatment for 28 days. Reactive oxygen species (ROS), antioxidant enzyme activities (glutathione peroxidase [GPx], superoxide dismutase [SOD], and catalase [CAT]), glutathione (GSH) levels, and oxidation markers (such as lipid peroxidation [malondialdehyde (MDA)] and protein oxidation [protein carbonyl (PCO)]) were analyzed in the kidney tissue. Additionally, renal fibrogenesis was studied through histopathological examination and the expression levels of fibrotic biomarkers.

**Results:**

The findings revealed that in vivo treatment with SiNPs significantly triggered oxidative stress in kidney tissues in a dose-dependent manner. This was characterized by increased production of ROS, elevated levels of MDA, PCO, and nitric oxide (NO), along with a significant decline in the activities of SOD, CAT, GPx, and reduced GSH. These changes were consistent with the histopathological analysis, which indicated interstitial fibrosis with mononuclear inflammatory cell aggregation, tubular degeneration, glomerulonephritis, and glomerular atrophy. The fibrosis index was confirmed using Masson's trichrome staining. Additionally, there was a significant upregulation of fibrosis-related genes, including transforming growth factor-beta 1 (TGF-β1), matrix metalloproteinases 2 and 9 (MMP-2/9), whereas the expression of tissue inhibitor of metalloproteinase 2 (TIMP2) was downregulated.

**Conclusions:**

This study provided a new research clue for the role of ROS and deregulated TGF-β signaling pathway in SiNPs nephrotoxicity.

## 1. Background

In recent years, nanoscience has experienced significant advancements due to the rapid expansion of nanotechnology and its broad applications (1). Nanotechnology is an emerging scientific field focused on synthesizing and developing materials within the nano-size range of 1 to 100 nm. Nanoparticles (NPs) have various applications in cosmetics, industry, agriculture, and medicine (2, 3). Due to their small size and high reactive surface, NPs can cross physiological barriers, enter the bloodstream, and target organs, thereby posing potential health risks (4, 5).

Silica NPs (SiNPs) rank among the top 5 engineered nanomaterials widely used in consumer goods, as listed by the Woodrow Wilson International Center for Scholars (6). SiNPs have found extensive application in various technological fields (7-9). Human exposure to SiNPs can occur through ingestion, inhalation, dermal contact, or direct injection into the systemic circulation via intraperitoneal or intravenous injection (10). Moreover, SiNPs exhibit high retention in the environment and food chain, which raises concerns regarding human exposure risks associated with their extensive usage (11).

In this context, both in vivo and in vitro studies have demonstrated the adverse effects of SiNPs on cellular components and cell morphology, leading to protein-DNA damage-induced apoptosis (12). Numerous studies have indicated that reactive oxygen species (ROS) play a role in SiNPs-induced hepatotoxicity (13), neurotoxicity (14), and cardiotoxicity (15).

Oxidative stress is recognized as a crucial molecular mechanism leading to fibrosis in various organs, including the liver and kidneys (16). Multiple mechanisms have been implicated in the pathogenesis of fibrosis (17).

Kidney fibrosis is the principal pathological process resulting from chronic renal damage, characterized by the accumulation and deposition of extracellular matrix (ECM). Matrix metalloproteinases (MMPs), a family of zinc-dependent endopeptidases, play a significant role in degrading ECM components and other non-ECM proteins such as cytokines and growth factors (18), while tissue inhibitors of metalloproteinases (TIMPs) inhibit collagen degradation. Alterations in the expression of MMPs and TIMPs can lead to the progression of fibrosis in pathological conditions (19).

Furthermore, inflammation, which is triggered by oxidative stress, is also believed to lead to the progression of fibrosis. Various factors, including increased production of ROS, can promote the production of a variety of cytokines and growth factors (20). Transforming growth factor-β1 (TGF-β1) is known to lead to the activation and proliferation of myofibroblasts in immune and vascular cells, leading to collagen accumulation. Elevated TGF-β expression in different tissues is associated with significant fibrotic changes. Hence, inflammation plays a crucial role in fibrosis induction. Currently, it remains unclear whether SiNPs lead to the progression of chronic kidney disease after intraperitoneal injection, and the underlying mechanisms are still unknown.

## 2. Objectives

Given the increasing biomedical applications of SiNPs, it is crucial to elucidate the nephrotoxicity associated with intraperitoneal exposure to SiNPs. While studies have shown the involvement of TGF-β signaling in SiNPs-induced fibrosis, it is evident that other mechanisms are also involved. Therefore, our objective was to investigate the potential kidney toxicity of SiNPs by examining oxidative stress, inflammation, fibrosis, and the underlying mechanisms involved.

## 3. Methods

### 3.1. Chemicals

We obtained commercially produced amorphous SiNPs (Silicon dioxide) with a primary size of 15 nm from Sigma-Aldrich Company (Deisenhofer, Germany). All reagents used in the study were purchased from Sigma-Aldrich (Deisenhofer, Germany), and they were of analytical grade.

### 3.2. Characterizations and Preparation of Silica Nanoparticle Suspension

SiNPs were characterized as described in our previous study (14). The spherical SiNPs had an average size of 11 ± 3 nm (14). To prepare the SiNPs for administration, they were resuspended in deionized water at 37°C and administered at concentrations of 25 and 100 mg SiNPs/kg/day (21). The SiNPs were dissolved and subjected to sonication using an ultrasonic device (Sonorex RK 52 H, Bandelin, Germany) for 15 minutes at 35% amplification. Fresh NP solutions were prepared daily immediately before treatment.

### 3.3. Animals and Treatments

This study used adult male Wistar rats, aged 8 - 9 weeks, with an average body weight ranging between 200 and 300 g. The rats were obtained from the Central Pharmacy (SIPHAT, Tunisia). The experimental procedures involving the animals were approved by the Ethical Committee of the Faculty of Sciences of Sfax-Tunisia, under protocol number 10.0768/20. The study was conducted in accordance with the general guidelines on the use of living animals in scientific investigations (Council of European Communities 1986) (22). All animals underwent a 1-week acclimatization period and were housed under the same laboratory conditions, with a temperature of 22 ± 2°C and a 12-h light/12-h dark cycle. They were provided with ad libitum access to food and water (23).

The rats were randomly divided into 3 groups (n = 10). The control group received only sterile water. The 2 experimental groups received a daily intraperitoneal injection of SiNPs at doses of 25 and 100 mg/kg for 28 consecutive days. The doses used in this study represent 1/200 and 1/50 of the median lethal dose (LD_50_) of SiNPs, respectively (21).

### 3.4. Collection and Preparation of Sample

At the end of the treatment period, all rats were sacrificed by cervical decapitation to minimize stress conditions. Blood samples were collected and centrifuged at 3000 rpm for 15 min at 4°C. Kidney samples were isolated, washed, and weighed. The kidney specimens were excised, placed in 10% formalin, and processed for histological assessment. Kidney sections were immersed in a phosphate buffer solution of 0.1 M (pH 7.4). Using a Teflon-glass homogenizer, the sections were homogenized on ice. The resulting homogenate was then centrifuged at 12000 rpm at 4°C for 15 min. The resulting supernatants were collected and used for further assays.

### 3.5. Assay of Oxidative Stress Markers and Antioxidants in the Kidney

The assessment of ROS in the homogenate samples was performed using 2′,7′-dichlorodihydrofluorescein diacetate (DCHF-DA), according to the method described by (24). The supernatant obtained from the homogenate was used to measure the levels of malondialdehyde (MDA) (25), protein carbonyl (PCO) contents (26), nitric oxide (NO) (27), hydrogen peroxide (H_2_O_2_) (28), catalase (CAT) activity (29), superoxide dismutase (SOD) activity (30), glutathione peroxidase (GPx) activity (31), reduced glutathione (GSH) concentration (32), and protein content (33).

### 3.6. Total RNA Isolation and Real-Time Reverse Transcriptase-Polymerase Chain Reaction Analysis

Total RNA was extracted according to the TRIzol manufacturer's protocol. The concentration and purity of each RNA sample were assessed by evaluating the DO_260_ and DO_260_/DO_280_ ratios using the NanoPhotometer™ (Implen, GmBH). The extracted RNA was then reverse transcribed into cDNA using the PrimerScript reverse transcriptase (TaKaRa). To evaluate the relative gene expression, a real-time polymerase chain reaction (PCR) was performed on a CFX96TM real-time PCR thermocycler (Biorad, France) using the SYBR® Green PCR Master Mix (TaKaRa). The 2^-ΔΔCt^ method was used to calculate the relative expression, with the β-actin gene serving as the reference standard. The primers used are listed in Table 1. 

**Table 1.** List of Primers Used in Quantitative Real-time Polymerase Chain Reaction Experiments

Gene Name
	Forward Primer	Reverse Primer
**β-actin**	5′-GAGATTACTGCCCT GGCTCCTA-3′	5′-GACTCATCGTACTCCTGCTTGCTG-3′
**TIMP-2 **	5′-AATTCGACTTGAAGTCTCAGAAGG-3′	5′-AAGTATTTGTCATGGCAGAAATAGG-3′
**TGF-β**	5′-GGGCTTTCGCTTCAGTGCT-3′	5′-TCGGTTCATGTCATGGATGGT-3′
**MMP-2**	5′-CGTGGTGAGATCTTCTTCTTCAAGGA-3′	5′-CCTCATACACAGCGTCAATCTTTTC-3′
**MMP-9**	5′-AATTCGACTTGAAGTCTCAGAAGG-3′	5′-AAGTATTTGTCATGGCAGAAATAGG-3′

Abbreviations: TIMP-2, tissue inhibitor of metalloproteinase 2; TGF-β, transforming growth factor-beta; MMP-2/9, matrix metalloproteinase 2/9.

### 3.7. Histopathology

Kidney tissues from both the normal and experimental rats were fixed in 10% buffered formalin and underwent standard processing for paraffin embedding. Sections with a thickness of 5 μm were deparaffinized, rehydrated, and stained using hematoxylin and eosin (H&E) solutions as well as Masson’s trichrome staining. The stained slides were observed under a Leica® microscope equipped with a Sony® digital camera to capture images for histological analysis. The sections were assessed for immune positivity and graded as follows: No (−), mild (+), moderate (++), and severe (+++).

### 3.8. Statistical Analysis

All data are presented as mean ± SEM for each group. Statistical significance between groups was determined using a one-way analysis of variance (ANOVA), followed by Tukey's post-hoc test for multiple comparisons. The level of significance was set at *P < 0.05, **P < 0.01, and ***P < 0.001 between the treated groups and controls.

## 4. Results

### 4.1. SiNPs Provoke Excessive Production of ROS and NO in the Kidneys of Rats

**Figure 1.**
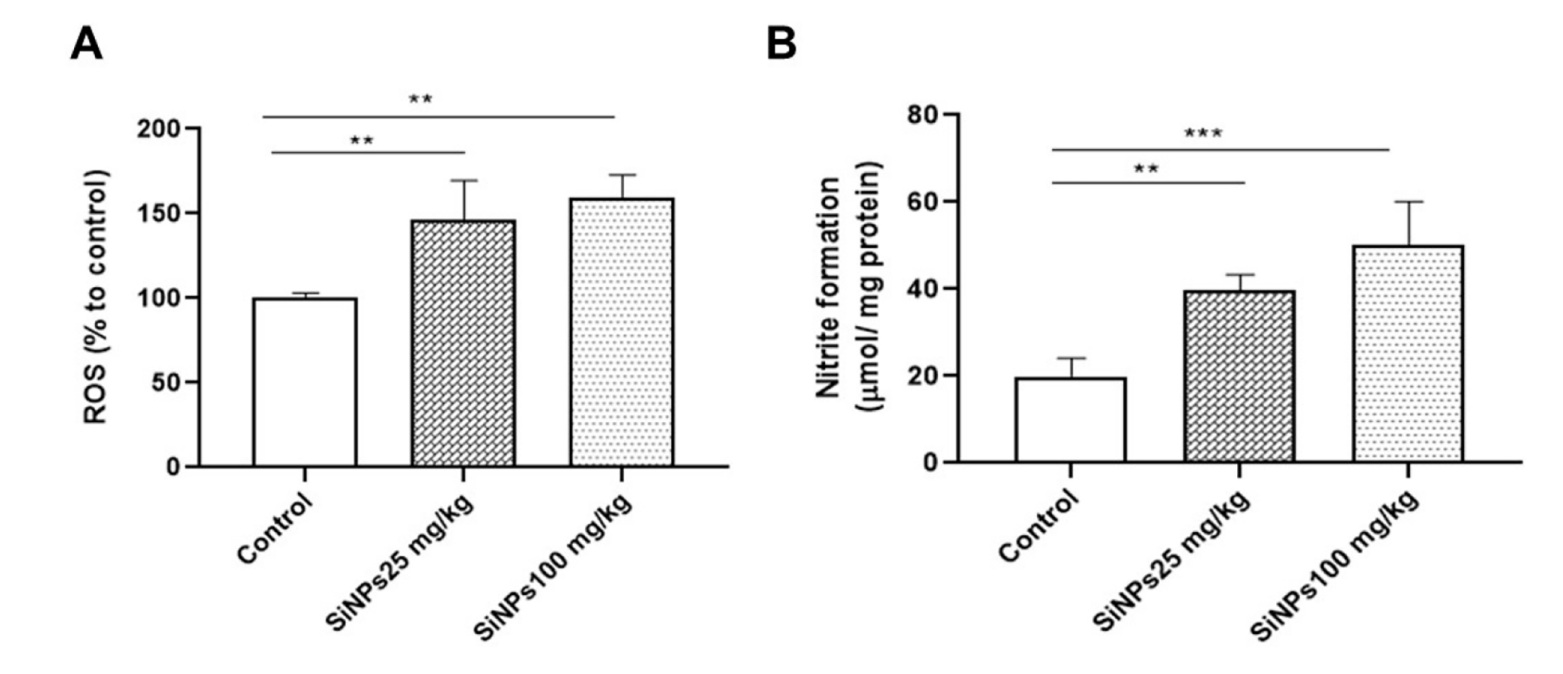
Silica nanoparticles (SiNPs) induced oxidative stress in the kidneys of rats at doses of 25 and 100 mg/kg of body weight for 28 days on the levels of A, reactive oxygen species and B, nitric oxide. Each value is expressed as the mean ± SEM of 10 rats per group. **P < 0.01 and ***P < 0.001: SiNPs groups compared to the control group. Abbreviations: ROS, reactive oxygen species; SiNPs, silica nanoparticles.

The levels of ROS were assessed using an H_2_DCF-DA fluorescent probe. Our results demonstrated a dose-dependent increase in ROS levels in the kidneys of rats administered with 25 and 100 mg/kg SiNPs for 28 days (Figure 1A). The administration of the lowest dose of SiNPs resulted in a significant increase in NO levels in the kidney (P < 0.01; Figure 1B) compared to the control group. Rats receiving 100 mg/kg SiNPs exhibited a significantly greater increase in NO levels (P < 0.001).

### 4.2. Silica Nanoparticles Provoke Oxidative Stress in the Kidney of Rats

Oxidative stress has been reported to be associated with kidney fibrosis (34). In this study, our hypothesis was that SiNPs induce oxidative stress in the kidney, leading to renal fibrosis. To validate this hypothesis, we examined the effects of SiNPs on 3 key indicators of oxidative stress: MDA, PCO, and H_2_O_2_. As shown in Table 2, rats receiving 25 mg/kg SiNPs exhibited non-significant changes (P > 0.05) in MDA levels compared to the control group, while significant increases (P < 0.05) were observed in PCO and H_2_O_2_ levels in renal tissue. On the other hand, exposure to 100 mg/kg SiNPs resulted in statistically significant elevations in MDA (P < 0.001), PCO (P < 0.01), and H_2_O_2_ (P < 0.01) levels, indicating tissue damage and the presence of oxidative stress. These results confirm the kidney toxicity and the induction of oxidative stress by SiNPs in rat kidneys following a 28-day exposure period.

**Table 2.** The Effect of Intraperitoneal Administration of Silica Nanoparticles at Doses of 25 and 100 mg/kg of Body Weight for 28 Days on Oxidative Stress Markers in the Kidney of Rats ^a^

Parameters	Groups		
	Control	25 mg SiNPs/kg	100 mg SiNPs/kg
**MDA (nmol/mg protein)**	1.375 ± 0.281	1.818 ± 0.123	2.801 ± 0.449 ***
**PCO (nmol/mg protein)**	21.898 ± 4.305	36.065 ± 9.322 *	48.647 ± 6.152 **
**H**_**2**_ **O**_**2**_ ** (nmol/mg protein)**	9.036 ± 3.145	25.804 ± 6.104 *	66.869 ± 11.056 **

Abbreviations: MDA, malondialdehyde; PCO, protein carbonyl; H_2_O_2_, hydrogen peroxide.

^a^ Each value is expressed as the mean ± SEM. N = 10 rats per group. * P < 0.05, ** P < 0.01, *** P < 0.001: Silica nanoparticle groups compared to the control group.

### 4.3. Silica Nanoparticles Suppress Antioxidant Defenses in the Kidneys of Rats

**Figure 2.**
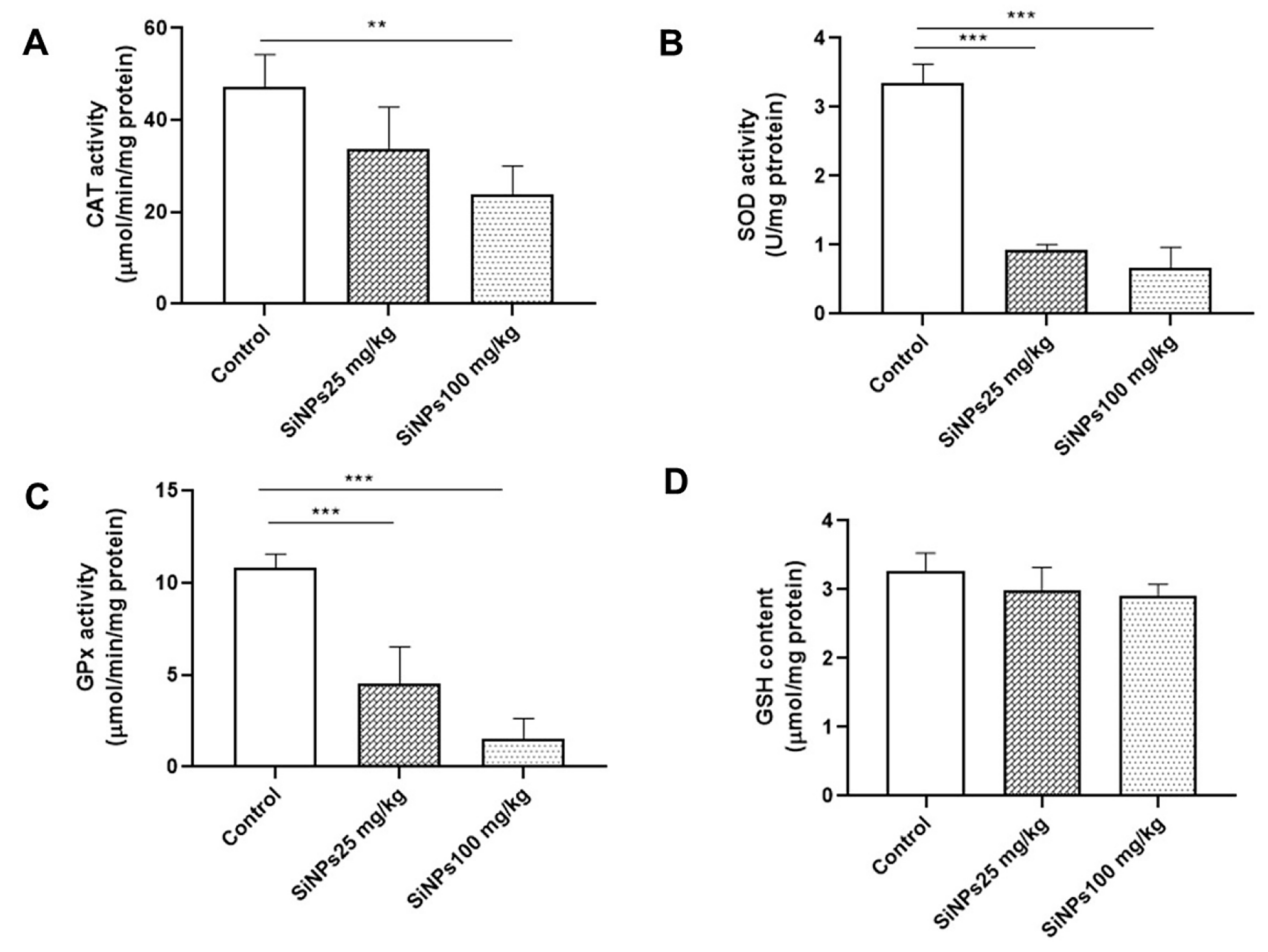
The effect of administration of silica nanoparticles (SiNPs) at different doses (25 and 100 mg/kg of body weight) for 28 days on renal enzymatic and non-enzymatic antioxidant levels. A, catalase activity (μmol/min/mg of proteins), B, superoxide dismutase (SOD) activity (USOD/mg of proteins), C, glutathione peroxidase (GPx) activity (μmol glutathione [GSH] consumed/min/mg of proteins), and D, GSH activity (µg/mg of protein). Each value is expressed as the mean ± SEM of 10 rats per group. *** P < 0.001, ** P < 0.01: SiNPs groups compared to the control group. Abbreviations: CAT, catalase; SOD, superoxide dismutase; GPx, glutathione peroxidase; GSH, glutathione.

To comprehensively investigate the impact of SiNPs on antioxidant defense, we assessed the GSH content and activities of the antioxidant enzymes SOD, CAT, and GPx in the kidney homogenate of rats exposed to SiNPs (Figure 2). SOD, CAT, and GPx are crucial antioxidant enzymes that play a vital role in combating oxidative stress and maintaining redox balance (35). Following intraperitoneal injection of SiNPs in rats, we observed reduced activities of SOD, CAT, and GPx at different doses compared to the control group (Figure 2A - C). However, when compared to the control group, rats exposed to different doses of 25 and 100 mg/kg SiNPs did not show a significant change in GSH content in the kidney (Figure 2D). 

### 4.4. Messenger RNA Expression of Fibrosis Factors in Kidney Tissue

**Figure 3.**
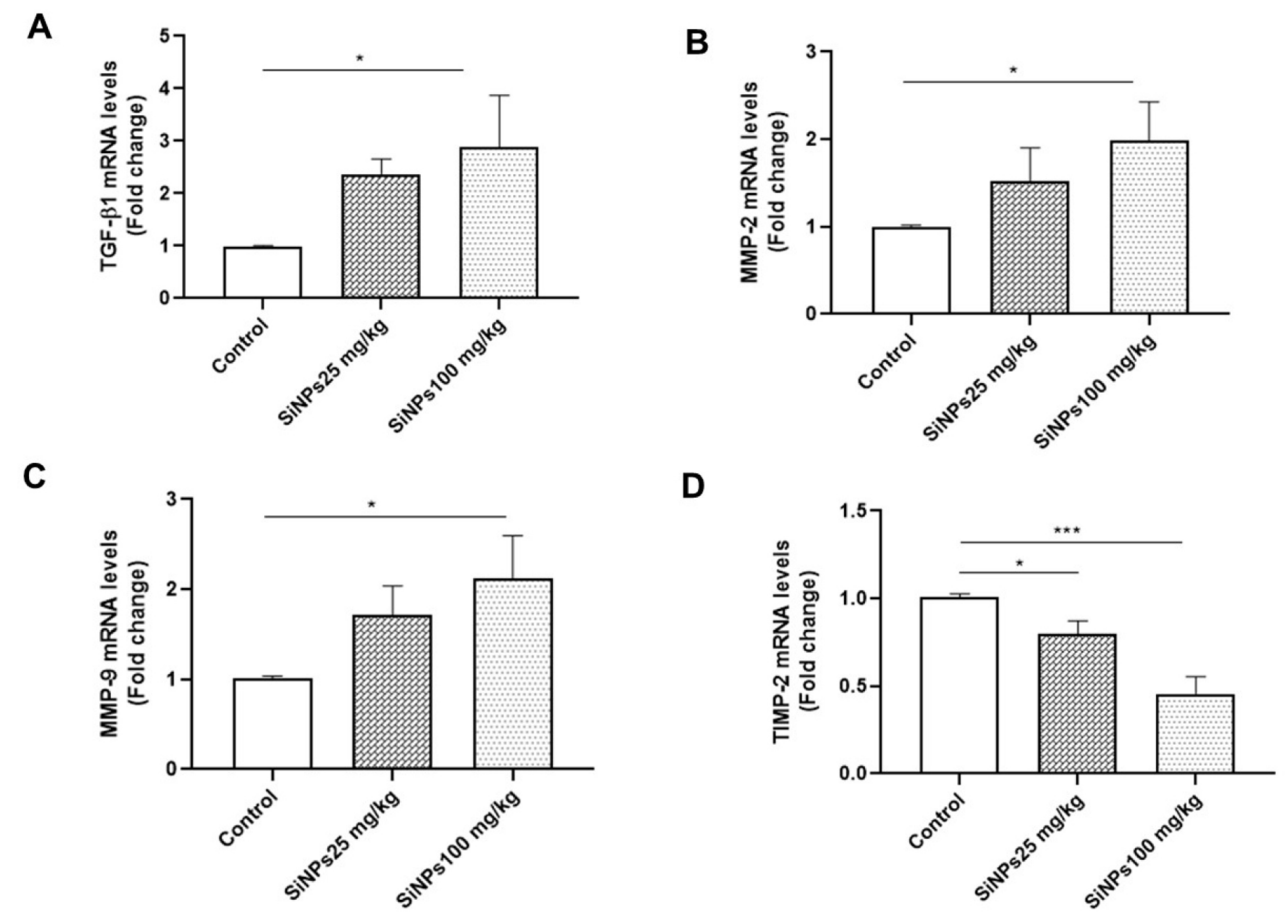
The effect of administration of silica nanoparticles (SiNPs) at doses of 25 and 100 mg/kg of body weight for 28 days on renal A, transforming growth factor-beta 1, B, matrix metalloproteinase 2 (MMP-2), C, MMP-9, and D, tissue inhibitor of metalloproteinase 2 expression levels. Each value is expressed as the mean ± SEM of 6 rats per group. *P < 0.05, ***P < 0.001: SiNPs groups compared to the control group. Abbreviations: TGF-β1, transforming growth factor-beta 1; MMP-2, matrix metalloproteinase 2; TIMP-2, tissue inhibitor of metalloproteinase 2.

Renal fibrosis is a common pathological feature and a final manifestation of chronic kidney diseases. To investigate whether SiNPs induce renal fibrosis, we determined the expression levels of MMP-9, MMP-2, TIMP-2, and TGF-β1 in kidney tissue using RT-qPCR. The results revealed a dose-dependent upregulation of MMP-9, MMP-2, and TGF-β1 expression following treatment with SiNPs, with significant differences observed in the 100-mg/kg SiNPs group compared to the control group (P < 0.05; Figure 3A - C). Furthermore, the mRNA expression levels of TIMP-2 showed significant dose-dependent downregulation in the 25- and 100-mg/kg SiNPs groups (P < 0.05 and P < 0.001, respectively; Figure 3D). 

### 4.5. Silica Nanoparticles Induce Kidney Fibrosis in Rats

**Figure 4.**
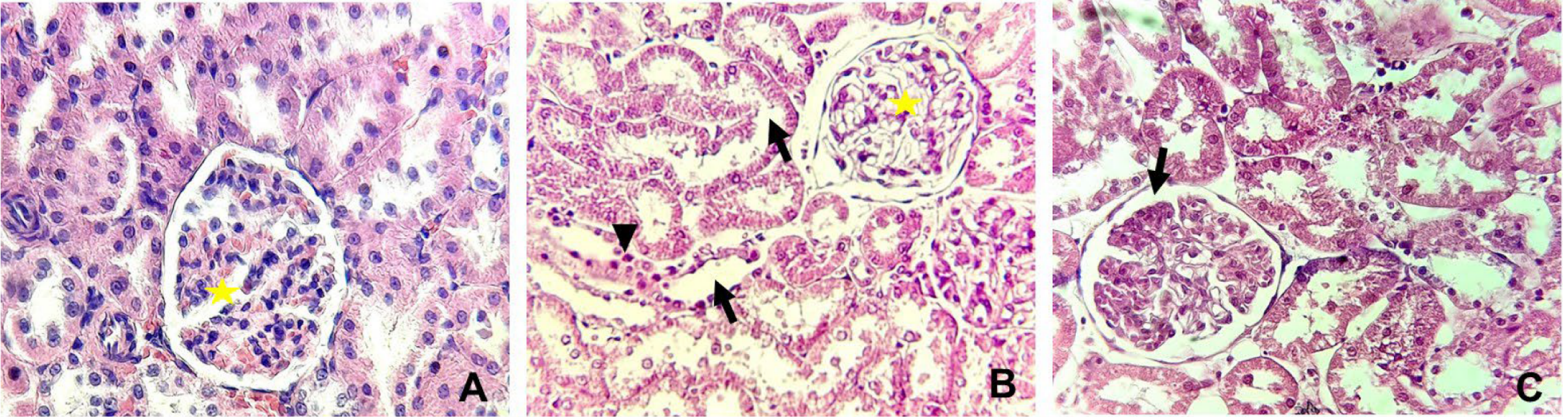
Histopathological alterations in the kidney in different groups examined by hematoxylin and eosin staining (X400). (A) The control group showing normal glomeruli (yellow star), (B) rats received 25 mg/kg of silica nanoparticles (SiNPs) showing marked interstitial fibrosis with mononuclear inflammatory cell aggregation (arrowhead), tubular degeneration (arrow), and glomerulonephritis (yellow star). In addition to these manifestations, (C) rats received 100 mg/kg of SiNPs showing glomerular atrophy (arrow).

**Figure 5.**
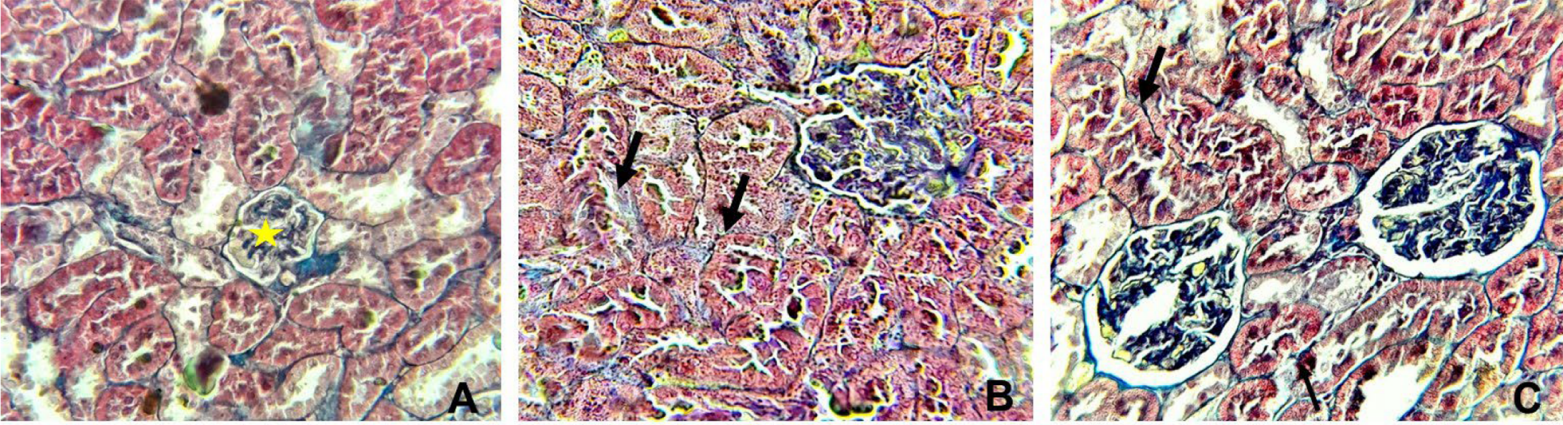
Histopathological alterations in the kidney in different groups examined by Masson’s trichrome (X400). A, the control group showing normal glomerular (yellow star), interstitial, and perivascular collagen, B, rats received 25 mg/kg of silica nanoparticles (SiNPs) showing mild interstitial fibrosis (arrow), and C, rats received 100 mg/kg of SiNPs showing marked interstitial fibrosis (arrow).

Table 3 and Figures 4 and 5 display the histopathological features of rat kidneys after 28 days of intraperitoneal exposure to different doses of SiNPs.

**Table 3.** Severity of Various Histopathological Alterations in Male Rats Exposed and Unexposed to Silica Nanoparticles ^a^

Variables	Control	SiNPs (mg/kg)	
		25	100
**Glomerulonephritis**	-	++	+++
**Glomerular atrophy**	-	-	+
**Tubular degeneration**	-	++	+++
**Interstitial inflammatory cell aggregates**	-	++	+++
**Leukocyte infiltration**	-	-	++

Abbreviation: SiNPs, silica nanoparticles.

^a^ (−) No change, (+) mild change, (++) moderate change, and (+++) severe change.

Microscopic examination of the control rat kidney stain was normal (Figures 4A and 5A). However, rats who received the lower dose of SiNPs (25 mg/kg of body weight) showed interstitial fibrosis with mononuclear inflammatory cell aggregation, tubular degeneration, and glomerulonephritis (Figures 4B and 5B). In addition to these manifestations, glomerular atrophy was observed in rats exposed to 100 mg/kg of SiNPs (Figures 4C and 5C).

## 5. Discussion

In this study, the administration of SiNPs at doses of 25 and 100 mg/kg of body weight to rats for 28 consecutive days resulted in nephrotoxicity characterized by severe oxidative stress, as indicated by a significant increase in MDA levels (a marker of lipid peroxidation), along with a significant decrease in GSH content and activities of antioxidant enzymes CAT, SOD, and GPx in the kidneys. Histopathological analysis of SiNPs-treated animals compared to the control group revealed interstitial fibrosis, mononuclear inflammatory cell aggregation, tubular degeneration, glomerulonephritis, and glomerular atrophy.

It is worth noting that NPs can reach various organs, including the kidney, liver, and spleen, through the circulatory system, irrespective of the route of exposure (36). The kidney, being the primary site for excretion, is particularly susceptible to the adverse effects of NP toxicity, which can impact renal function (37).

Our findings suggest that SiNPs may induce oxidative stress through a ROS-mediated process. The unsaturated surface bonds and hydroxyl groups on the silica surface contribute to oxidative damage and the generation of ROS (38).

The interaction of NPs, including SiNPs, with cells disrupts the balance between prooxidants and antioxidants, leading to mitochondrial dysfunction, inhibition of enzyme activity, and cell death due to DNA damage (39, 40).

Consistent with our previous study, SiNPs-induced liver and brain damage was demonstrated to be attributed to oxidative stress induction, followed by lipid peroxidation, inflammation, and apoptosis, which impair their functions (13, 14).

Oxidative stress, which is pathologically evident in certain diseases, plays a crucial role in the development of fibrogenesis under pathological conditions (41). To explore potential mechanisms of renal fibrosis, we measured the content of key renal fibrotic markers (TGF-β, TIMP, and MMP-2/9) using real-time PCR. Our results indicated an upregulation of MMP-2/9 and TGF-β1, coupled with a downregulation of TIMP-2 in the SiNPs-exposed groups. Reactive oxygen species can modulate TGF-β signaling by enhancing its expression and activation. TGF-β is a potent and widespread profibrogenic cytokine that plays a significant role in the development of fibrosis and leads to extensive deposition of ECM in various organs, such as the heart, lungs, kidneys, and liver (42). TGF-β1 is a critical profibrotic cytokine and is considered a central mediator in the pathogenesis of renal fibrosis. According to Tang et al. (43), TGF-β expression is upregulated in animal models of renal fibrosis, as well as in human counterparts.

Similarly, MMP-9) plays a profibrotic role in renal fibrosis (44). It promotes the epithelial-mesenchymal transition of epithelial tubular cells, which is associated with renal fibrosis in both proximal tubular and glomerular epithelial cells (45).

Wan et al. (46) reported that certain NPs can disrupt the balance between (MMPs and their tissue inhibitors, leading to MMP overexpression. Based on this, we hypothesize that the upregulation of MMP-2 and MMP-9 induced by SiNPs results in the downregulation of TIMPs, thereby inhibiting the action of MMPs. The activation of MMPs is tightly regulated by their endogenous inhibitors, TIMPs. Our findings show that SiNP exposure downregulates the expression of TIMP-2, suggesting that ROS production, as observed in previous studies, also plays a role in the regulation of TIMP-2.

Reactive oxygen species have been shown to activate latent MMPs in conditioned media (47). Analysis of the promoters of MMPs and TIMPs has revealed the presence of other elements, including activator protein-1 (AP-1) elements and multiple Ets elements (48).

**Figure 6.**
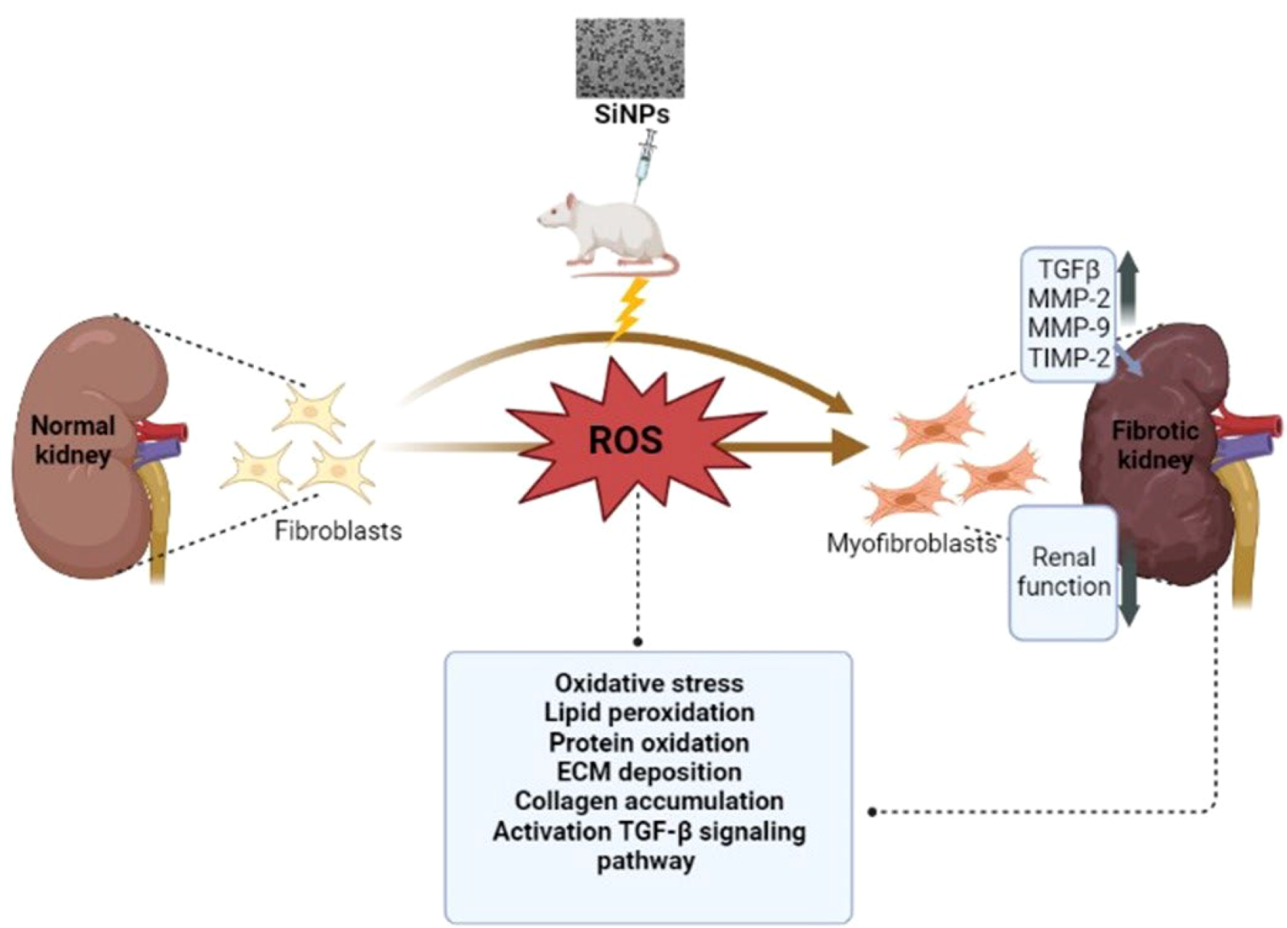
A proposed schematic diagram illustrating silica nanoparticles-induced renal toxicity and fibrosis.

In summary, the data from this study lead us to conclude that exposure to SiNPs has toxic effects on the kidneys of male rats (Figure 6). 

### 5.1. Conclusions

This study provides insights into the altered signaling pathways in the kidney resulting from intraperitoneal exposure to SiNPs. This includes the upregulation of numerous marker genes involved in the fibrosis process and an imbalance between antioxidant defenses and the production of ROS. While this study offers an overview of the significant biochemical, molecular, and histopathological changes induced by SiNPs in the kidney, it is important to note that other endpoints and signaling pathways may also play a crucial role, requiring further research. Collectively, the results of the present study provide an understanding of the mechanistic pathways activated by SiNPs to enhance the nephrotoxic effect.

## Data Availability

All data is provided in the manuscript and in additional files. All data are available on request.

## References

[1] Ghareeb S, Ragheb D, El-Sheakh A, Ashour MA (2022). Potential Toxic Effects of Exposure to Titanium Silicon Oxide Nanoparticles in Male Rats.. Int J Environ Res Public Health..

[2] Boyes WK, Chen R, Chen C, Yokel RA (2012). The neurotoxic potential of engineered nanomaterials.. Neurotoxicology..

[3] Zhu S, Gong L, Li Y, Xu H, Gu Z, Zhao Y (2019). Safety Assessment of Nanomaterials to Eyes: An Important but Neglected Issue.. Adv Sci (Weinh)..

[4] Abbott LC, Maynard AD (2010). Exposure assessment approaches for engineered nanomaterials.. Risk Anal..

[5] Ghonimi WAM, Alferah MAZ, Dahran N, El-Shetry ES (2022). Hepatic and renal toxicity following the injection of copper oxide nanoparticles (CuO NPs) in mature male Westar rats: histochemical and caspase 3 immunohistochemical reactivities.. Environ Sci Pollut Res Int..

[6] Vance ME, Kuiken T, Vejerano EP, McGinnis SP, Hochella MF, Rejeski D (2015). Nanotechnology in the real world: Redeveloping the nanomaterial consumer products inventory.. Beilstein J Nanotechnol..

[7] Wang Y, Song H, Yu M, Xu C, Liu Y, Tang J (2018). Room temperature synthesis of dendritic mesoporous silica nanoparticles with small sizes and enhanced mRNA delivery performance.. J Mater Chem B..

[8] Diao J, Xia Y, Jiang X, Qiu J, Cheng S, Su J (2021). Silicon dioxide nanoparticles induced neurobehavioral impairments by disrupting microbiota-gut-brain axis.. J Nanobiotechnology..

[9] Li H, Zhu J, Xu YW, Mou FF, Shan XL, Wang QL (2022). Notoginsenoside R1-loaded mesoporous silica nanoparticles targeting the site of injury through inflammatory cells improves heart repair after myocardial infarction.. Redox Biol..

[10] Guo C, Liu Y, Li Y (2021). Adverse effects of amorphous silica nanoparticles: Focus on human cardiovascular health.. J Hazard Mater..

[11] Murugadoss S, Lison D, Godderis L, Van Den Brule S, Mast J, Brassinne F (2017). Toxicology of silica nanoparticles: an update.. Arch Toxicol..

[12] Yang Y, Du X, Wang Q, Liu J, Zhang E, Sai L (2019). Mechanism of cell death induced by silica nanoparticles in hepatocyte cells is by apoptosis.. Int J Mol Med..

[13] Aouey B, Boukholda K, Gargouri B, Bhatia HS, Attaai A, Kebieche M (2022). Silica Nanoparticles Induce Hepatotoxicity by Triggering Oxidative Damage, Apoptosis, and Bax-Bcl2 Signaling Pathway.. Biol Trace Elem Res..

[14] Boukholda K, Gargouri B, Aouey B, Attaai A, Elkodous MA, Najimi M (2021). Subacute silica nanoparticle exposure induced oxidative stress and inflammation in rat hippocampus combined with disruption of cholinergic system and behavioral functions.. NanoImpact..

[15] Guerrero-Beltran CE, Bernal-Ramirez J, Lozano O, Oropeza-Almazan Y, Castillo EC, Garza JR (2017). Silica nanoparticles induce cardiotoxicity interfering with energetic status and Ca(2+) handling in adult rat cardiomyocytes.. Am J Physiol Heart Circ Physiol..

[16] Mahmoud AM, Desouky EM, Hozayen WG, Bin-Jumah M, El-Nahass ES, Soliman HA (2019). Mesoporous Silica Nanoparticles Trigger Liver and Kidney Injury and Fibrosis Via Altering TLR4/NF-kappaB, JAK2/STAT3 and Nrf2/HO-1 Signaling in Rats.. Biomolecules..

[17] Wynn TA, Ramalingam TR (2012). Mechanisms of fibrosis: therapeutic translation for fibrotic disease.. Nat Med..

[18] Wang X, Khalil RA (2018). Matrix Metalloproteinases, Vascular Remodeling, and Vascular Disease.. Adv Pharmacol..

[19] Robert S, Gicquel T, Victoni T, Valenca S, Barreto E, Bailly-Maitre B (2016). Involvement of matrix metalloproteinases (MMPs) and inflammasome pathway in molecular mechanisms of fibrosis.. Biosci Rep..

[20] Babalola O, Mamalis A, Lev-Tov H, Jagdeo J (2014). NADPH oxidase enzymes in skin fibrosis: molecular targets and therapeutic agents.. Arch Dermatol Res..

[21] van der Zande M, Vandebriel RJ, Groot MJ, Kramer E, Herrera Rivera ZE, Rasmussen K (2014). Sub-chronic toxicity study in rats orally exposed to nanostructured silica.. Part Fibre Toxicol..

[22] Council of European Communities (1986). Council Directive 86/609/EEC of 24 November 1986 on the approximation of laws, regulations and administrative provisions of the Member States regarding the protection of animals used for experimental and other scientific purposes.. Official Journal of the European Communities..

[23] Fetoui H, Mahjoubi-Samet A, Jammousi K, Ellouze F, Guermazi F, Zeghal N (2006). Energy restriction in pregnant and lactating rats lowers bone mass of their progeny.. Nutrition Research..

[24] Driver AS, Kodavanti PR, Mundy WR (2000). Age-related changes in reactive oxygen species production in rat brain homogenates.. Neurotoxicol Teratol..

[25] Draper HH, Hadley M (1990). Malondialdehyde determination as index of lipid peroxidation.. Methods Enzymol..

[26] Reznick AZ, Packer L (1994). Oxidative damage to proteins: spectrophotometric method for carbonyl assay.. Methods Enzymol..

[27] Green LC, Wagner DA, Glogowski J, Skipper PL, Wishnok JS, Tannenbaum SR (1982). Analysis of nitrate, nitrite, and [15N]nitrate in biological fluids.. Anal Biochem..

[28] Gay C, Collins J, Gebicki JM (1999). Determination of iron in solutions with the ferric-xylenol orange complex.. Anal Biochem..

[29] Aebi H (1984). Catalase in vitro.. Methods Enzymol..

[30] Marklund S, Marklund G (1974). Involvement of the superoxide anion radical in the autoxidation of pyrogallol and a convenient assay for superoxide dismutase.. Eur J Biochem..

[31] Flohe L, Gunzler WA (1984). Assays of glutathione peroxidase.. Methods Enzymol..

[32] Ellman GL (1959). Tissue sulfhydryl groups.. Arch Biochem Biophys..

[33] Bradford MM (1976). A rapid and sensitive method for the quantitation of microgram quantities of protein utilizing the principle of protein-dye binding.. Anal Biochem..

[34] Su H, Wan C, Song A, Qiu Y, Xiong W, Zhang C (2019). Oxidative Stress and Renal Fibrosis: Mechanisms and Therapies.. Adv Exp Med Biol..

[35] Kuyumcu F, Aycan A (2018). Evaluation of Oxidative Stress Levels and Antioxidant Enzyme Activities in Burst Fractures.. Med Sci Monit..

[36] Najahi-Missaoui W, Arnold RD, Cummings BS (2020). Safe Nanoparticles: Are We There Yet?. Int J Mol Sci..

[37] Suresh Vs R (2021). Recent Advances on Renal Toxicity of Engineered Nanoparticles-A Review.. J Toxicol Risk Assess..

[38] Napierska D, Thomassen LC, Lison D, Martens JA, Hoet PH (2010). The nanosilica hazard: another variable entity.. Part Fibre Toxicol..

[39] Guo C, Sun L, Chen X, Zhang D (2013). Oxidative stress, mitochondrial damage and neurodegenerative diseases.. Neural Regen Res..

[40] Juan CA, Perez de la Lastra JM, Plou FJ, Perez-Lebena E (2021). The Chemistry of Reactive Oxygen Species (ROS) Revisited: Outlining Their Role in Biological Macromolecules (DNA, Lipids and Proteins) and Induced Pathologies.. Int J Mol Sci..

[41] Cheresh P, Kim SJ, Tulasiram S, Kamp DW (2013). Oxidative stress and pulmonary fibrosis.. Biochim Biophys Acta..

[42] Richter K, Konzack A, Pihlajaniemi T, Heljasvaara R, Kietzmann T (2015). Redox-fibrosis: Impact of TGFbeta1 on ROS generators, mediators and functional consequences.. Redox Biol..

[43] Tang F, Hao Y, Zhang X, Qin J (2017). Effect of echinacoside on kidney fibrosis by inhibition of TGF-beta1/Smads signaling pathway in the db/db mice model of diabetic nephropathy.. Drug Des Devel Ther..

[44] Tan TK, Zheng G, Hsu TT, Lee SR, Zhang J, Zhao Y (2013). Matrix metalloproteinase-9 of tubular and macrophage origin contributes to the pathogenesis of renal fibrosis via macrophage recruitment through osteopontin cleavage.. Lab Invest..

[45] Fragiadaki M, Mason RM (2011). Epithelial-mesenchymal transition in renal fibrosis - evidence for and against.. Int J Exp Pathol..

[46] Wan R, Mo Y, Zhang X, Chien S, Tollerud DJ, Zhang Q (2008). Matrix metalloproteinase-2 and -9 are induced differently by metal nanoparticles in human monocytes: The role of oxidative stress and protein tyrosine kinase activation.. Toxicol Appl Pharmacol..

[47] Gasche Y, Copin JC, Sugawara T, Fujimura M, Chan PH (2001). Matrix metalloproteinase inhibition prevents oxidative stress-associated blood-brain barrier disruption after transient focal cerebral ischemia.. J Cereb Blood Flow Metab..

[48] Clark IM, Swingler TE, Sampieri CL, Edwards DR (2008). The regulation of matrix metalloproteinases and their inhibitors.. Int J Biochem Cell Biol..

